# Toxic but Drank: Gustatory Aversive Compounds Induce Post-ingestional Malaise in Harnessed Honeybees

**DOI:** 10.1371/journal.pone.0015000

**Published:** 2010-10-27

**Authors:** Ainara Ayestaran, Martin Giurfa, María Gabriela de Brito Sanchez

**Affiliations:** 1 Université de Toulouse (UPS), Centre de Recherches sur la Cognition Animale, Toulouse, France; 2 Centre National de la Recherche Scientifique (CNRS), Centre de Recherches sur la Cognition Animale, Toulouse, France; Université Pierre et Marie Curie, France

## Abstract

**Background:**

Deterrent substances produced by plants are relevant due to their potential toxicity. The fact that most of these substances have an unpalatable taste for humans and other mammals contrasts with the fact that honeybees do not reject them in the range of concentrations in which these compounds are present in flower nectars. Here we asked whether honeybees detect and ingest deterrent substances and whether these substances are really toxic to them.

**Results:**

We show that pairing aversive substances with an odor retards learning of this odor when it is subsequently paired with sucrose. Harnessed honeybees in the laboratory ingest without reluctance a considerable volume (20 µl) of various aversive substances, even if some of them induce significant post-ingestional mortality. These substances do not seem, therefore, to be unpalatable to harnessed bees but induce a malaise-like state that in some cases results in death. Consistently with this finding, bees learning that one odor is associated with sugar, and experiencing in a subsequent phase that the sugar was paired with 20 µl of an aversive substance (devaluation phase), respond less than control bees to the odor and the sugar. Such stimulus devaluation can be accounted for by the malaise-like state induced by the aversive substances.

**Conclusion:**

Our results indicate that substances that taste bitter to humans as well as concentrated saline solutions base their aversive effect on the physiological consequences that their ingestion generates in harnessed bees rather than on an unpalatable taste. This conclusion is only valid for harnessed bees in the laboratory as freely-moving bees might react differently to aversive compounds could actively reject aversive substances. Our results open a new possibility to study conditioned taste aversion based on post-ingestional malaise and thus broaden the spectrum of aversive learning protocols available in honeybees.

## Introduction

Bitter substances are biologically relevant due to their potential toxicity. It is therefore not surprising that mammals have specialized cells to detect them [1]–[2]. Bitter substances are also important in insect-plant relationships because plants may use these substances for protection against herbivores and insect pests [3]–[4]. In the case of insects, however, the term ‘bitter’, which is associated with a specific human sensation, constitutes an anthropocentrism. It is, therefore, more appropriate to refer to these substances as ‘deterrent’ or ‘aversive’, which allows extending the use of the term to non-bitter compounds which could also exert a repelling action on insects [5]–[6].

Taste perception in insects occurs through gustatory receptor neurons (Grns) located within gustatory hairs or sensillae located on different body appendages. Generally, these sensillae also host a mechanoreceptor neuron which conveys information on mechanical bending upon surface contact. Grns are tuned to different tastants because they present on their membrane different kinds of molecular gustatory receptors (Grs), i.e. molecular structures allowing the binding of specific chemicals. Dedicated molecular receptors for substances which are bitter for humans have been identified in the fruit fly *Drosophila melanogaster*, the insect in which most studies on taste perception have been so far performed [7]. In this insect, 68 Grs encoded by 60 genes through alternative splicing have been identified [8]–[11]. Two of these Grs, DmGr66a and DmGr93a, have been associated with “bitter” taste as they both respond to caffeine and are coexpressed in the same Grns [12]–[14].

Similar studies performed in other insects have yielded comparable results. Both in the yellow-fever mosquito *Aedes aegypti* [15] and the malaria mosquito *Anopheles gambiae* [16], Grs similar to DmGr66a have been identified (AaGr14 and AgGr2, respectively). Given the fact that both are dipterans, similarities with the fruit fly can be understood. In a non-related insect, the silk moth *Bombyx mori*, most of their 66 Grs belong to a large gene subfamily expansion that might constitute their “bitter” taste receptors [17]. Also, in the pea aphid *Acyrthosiphon pisum*, two Gr subfamily expansions of 21 and 41 genes might represent “bitter” taste receptors. [18]. These insects directly feed on foliage and sap so that detecting plant defensive compounds through ‘bitter’ receptor would be relevant to them. Different results have been found for the honeybee *Apis mellifera*. The decoding of the genome of this insect [19] yielded a surprising result: only 10 Grs seem to exist in the honeybee [20] so that its gustatory world might be described as being poor. Among these receptors none is similar to DmGr66a or DmGr93a, thus raising the question of whether honeybees can detect bitter substances at all.

“Bitter” perception in honeybees has received so far little attention [21]. Electrophysiological recordings of Grns located within taste sensillae of the antennae [6], mouthparts and tarsi [22] were unable to find neurons responding to different kinds of aversive gustatory tastants. Furthermore, behavioral experiments on harnessed bees stimulated on the antennae [6] and the tarsi [22] showed that bees did not exhibit strong rejection when stimulated with aversive gustatory substances such as quinine and salicine. Although these results are consistent with the evidence provided by molecular studies on Grs (see above), they are intriguing because, in a natural context, bees may be confronted with aversive substances present in pollen, nectar [23]–[26], and resins collected for the elaboration of propolis [27]. Yet, the contents of aversive compound in the natural products exploited by bees are usually low so that the behavior of bees is generally unaffected [26].

The double question of whether honeybees detect and ingest deterrent substances and whether these substances are really toxic to them has not been explicitly answered so far. If bees possess the faculty of detecting deterrent compounds though their unpalatable taste, they should strongly reject them. Conversely, if bees are unable to sense these compounds, they may ingest them and experience, as a consequence of their toxicity, a subsequent malaise that may lead to rejection of the plants producing this undesirable after-effect. In both cases, the result would be similar as bees would learn to avoid the toxic sources, but the behavioral and physiological pathways leading to this result would be different.

Although in natural situations, bees might never experience aversive substances at concentrations high enough to exert a malaise on them [26], the question of whether or not important quantities of these substances are ingested by these insects is interesting given contradictory reports on this point. On one hand, harnessed honeybees in the laboratory do not exhibit obvious rejection of highly concentrated aversive compounds such as quinine or salicine [6]. On the other hand, free-flying bees learn better visual discriminations if visual distracters are associated with quinine thus indicating that this substance would exert an aversive, penalizing effect [28]–[30].

Here we aimed at determining whether deterrent substances delivered to the mouthparts of harnessed honeybees are rejected or ingested, and whether the eventual ingestion of these compounds induces a state of malaise that may affect *a posteriori* stimulus evaluation. We performed experiments in which we determined survival probability following bitter compound ingestion and learning experiments in which we studied the effect of bitter compounds on acquisition and stimulus devaluation. Our final goal was, therefore, to provide novel insights into the gustatory world of honeybees by focusing on the effects of deterrent tastants, many of which are perceived as bitter by humans.

## Materials and Methods

Honeybees, *Apis mellifera*, from a hive located at 50 m from the laboratory were caught in the morning, placed in glass vials, and cooled down on ice until they stopped moving. They were then harnessed in individual small tubes so that they could only move their antennae and mouthparts, including the proboscis. Bees were then fed with sucrose solution 1 M and kept in the dark and in high humidity for approximately two and half hours. All chemicals used were from Sigma – Aldrich (France).

We performed three series of experiments. In ***Experiment 1***, we studied whether pairing an odor with a deterrent substance in a 1^st^ pre-exposure phase determines a retardation of olfactory learning in a 2^nd^ conditioning phase in which the same odor is now paired with the appetitive reward of sucrose [31]. If deterrent substances exert an aversive effect in bees, they should confer a negative associative strength to the odorant paired with them so that it would be difficult to revert this learning in a subsequent phase. In ***Experiment 2***, we asked whether bees drink a considerable amount of deterrent substances (20 µl, i.e. a third of their crop capacity) [32] and studied the mortality resulting from this ingestion. Finally, in ***Experiment 3***, we analyzed whether bees having learned that and odor is followed by sugar in a 1^st^ phase, and associating afterwards that this sugar is paired with a deterrent substance in a 2^nd^ phase, ‘devaluate’ the sugar and the conditioned odor, thus exhibiting a reduced responsiveness to these stimuli (odor and sugar) in a 3^rd^ phase [33]. Such devaluation, if any, would reveal that the substances delivered in the 2^nd^ phase have a true aversive nature.

### Experiment 1

In a pre-exposure phase, we trained bees to associate 1-nonanol with different reinforcements, including deterrent substances; in a second conditioning phase, we trained the same bees to associate 1-nonanol with sucrose 1 M. Six groups of bees were used. Groups differed in the treatment received in the pre-exposure phase but experienced all the same appetitive conditioning in the second phase.

Each subject was checked for intact appetitive responses to sucrose before starting the 1^st^ and 2^nd^ phases. This was done by lightly touching the antennae with a toothpick soaked with sucrose solution 1 M without subsequent feeding. This stimulation elicits the appetitive proboscis extension reflex (PER), which is an unconditioned response to sucrose [34]–[36]. Extension of the proboscis beyond a virtual line between the open mandibles was counted as PER. Animals that did not show the reflex to sucrose before conditioning were discarded. During conditioning, gustatory stimuli were delivered to the proboscis tip by means of a toothpick soaked in the solution tested. If a substance different from sucrose was unable to elicit PER, the proboscis was gently extended with the toothpick and the solution was then delivered to the tip of the proboscis.

In the ***first pre-exposure phase***, bees received four pairings of 1-nonanol with either distilled water (water group), NaCl 3 M (NaCl group), quinine 100 mM (quinine group), salicine 100 mM (salicine group) or a mechanical stimulation of antennae and proboscis with a dry toothpick (mechanosensory group). A sixth group was left untreated (untreated group). Quinine and salicine solutions were highly concentrated to potentiate their eventual aversive effect. Highly concentrated NaCl was also used as it is considered to be an aversive reinforcement in olfactory PER conditioning [37]. Distilled water is tasteless and provides a gustatory control. The mechanosensory group, on the other hand, allows appreciating the contribution of the mechanosensory stimulation induced by the toothpick. In all cases trials were separated by 10 min.

In the ***second conditioning phase***, all six groups, including the one that received no treatment in the first phase, experienced four pairings of 1-nonanol with sucrose solution 1 M. Trials were separated by 10 min. The two phases were also separated by 10 min.

In both phases, trials lasted 1 min. The bee harnessed in its individual tube was placed in an experimental holder with an air extractor placed behind it for 25 sec to allow familiarization with the setup. The air extractor impeded the accumulation of residual odors. Thereafter, the odorant 1-nonanol (conditioned stimulus or CS) was released for 6 sec. Three sec after CS onset, the bee got its antennae and proboscis stimulated with its particular treatment during 6 sec (water, quinine, salicine, NaCl, or mechanosensory stimulation in the pre-exposure phase; sucrose 1 M in the conditioning phase; see above). Both stimulus overlap and interstimulus interval were therefore 3 sec. The bee was left in the conditioning place during 29 sec and then removed.

In both phases, we recorded PER to 1-nonanol (conditioned responses). Multiple responses during a single stimulation were counted as a single PER. The percentage of PER recorded was used to represent acquisition curves. Analysis of variance (ANOVAs) for repeated measurements was used both for between-group and for within-group comparisons. Monte Carlo studies have shown that it is permissible to use ANOVA on dichotomous data under controlled conditions [37], which are met by our experiments (equal cell frequencies and at least 40 degrees of freedom of the error term).

### Experiment 2

We determined the probability of survival of harnessed honeybees following feeding of aversive compounds. We quantified the number of dead bees at 60, 90, 120, 180 and 240 min following feeding of the last bee in a group. Different groups of bees were fed with different substances. Within each group, each harnessed bee was fed 20 µl (4 times 5 µl; i.e. one third of their full crop load) [32] of the aversive (or control) substance assigned to its group. A graded micropipette was used to feed the bees so that we could verify the amount of solution ingested by the bees.

Two experimental series were performed following this procedure: in a *first series*, we used the following solutions: distilled water (water group), NaCl 3 M (NaCl group), salicine 100 mM (salicine group) and quinine 100 mM (quinine group); in a *second series*, we used distilled water (water group), sucrose 1 M (sucrose group), quinine 10 mM and 100 mM (quinine 10 and quinine 100 groups), lithium chloride 140 mM (LiCl group), amygdalin 1 mM (amygdalin group), L-canavanine 40 mM and 100 mM (L-canavanine 40 and L-canavanine 100 groups), a mixture of quinine 10 mM and sucrose 1 M (quinine + sucrose group) and a mixture of LiCl 140 mM and sucrose 1 M (LiCl + sucrose group).

Solutions chosen for the *first series* correspond to those used in Experiment 1 (see above). Those employed in the *second series* were aimed at increasing the spectrum of aversive substances tested. In this series, we included, for instance, a cyanogenic glycoside, amygdalin, which has been shown to reduced food intake in two noctuid caterpillars [39]; its concentration (1 mM) was chosen based on the work of Singaravelan *et al.* [26] who found that a concentration of 0.1 mM did not induce any behavioral effect in bees; we thus increased the concentration in one order of magnitude to detect such an effect, if any. We also employed L-canavanine as it is a highly toxic L-arginine analog present in plants. The concentration used (40 mM) corresponds to that exerting an aversive effect in the fruit fly [40]; that of 100 mM was obtained by extrapolating the lethal dose 50% (DL50) for rats to the average weight of honeybees. We also included LiCl which is a salt that induces malaise and nausea in rodents; the concentration of 140 mM corresponds again to the mouse's DL50 extrapolated to honeybees. Water and sucrose 1 M solutions acted as controls. While bees fed with water can exhibit a basal mortality during the time due to exhaustion and lack of energetic resources, bees fed with sucrose solution should exhibit no or less mortality. In this series we used again quinine, but we compared the effects of a highly concentrated (100 mM) and a diluted quinine solution (10 mM). Finally we fed the bees with mixtures of quinine 10 mM and sucrose 1 M, or LiCl 140 mM and sucrose 1 M, to determine whether quinine and LiCl suppress sucrose perception in a mixture; such an effect has been reported for quinine and sucrose in electrophysiological recordings of antennal gustatory sensillae [6].

In both series, survival analysis was performed using as censored observations the individuals that survived at the end of the measuring period [41]. For each treatment (i.e. solution fed), we computed the cumulative proportion of surviving and established Kaplan-Meier's survival functions defined as the probability of surviving at least to time t. We used a log rank test to compare multiple samples, which is a standard procedure in survival analyses [38]. Such a log rank test follows a χ^2^ distribution in the case of multiple-sample comparison; in the case of two-sample comparisons, it computes a Z score referred to a normal distribution.

### Experiment 3

We used the logic of reinforcer-devaluation experiments [33] to determine whether aversive substances induce a change in responsiveness to sucrose and to a previously conditioned odor in bees. In this kind of experiments, animals first receive a CS –US conditioning, and afterwards, the value of the US is altered in absence of the CS. To this end, after CS-US conditioning, the US is paired with an aversive treatment (like an aversive gustatory substance or injection of a substance inducing malaise). Post conditioning treatments like associating the US with an unpleasant taste or with malaise are thought to devaluate the US representation (an effect termed US devaluation) [33], [42]. The logic underlying this procedure is that if the stimulus representation of the US were associated with that of the CS, then changes in the value of the US would also alter responding to the CS.

This protocol is interesting because if some of the substances used in previous experiments have an un pleasant taste, pairing sugar with them after an odor-sugar conditioning would lead to sugar devaluation, and in consequence to the devaluation of the odor itself. In this case a significant decrease in responses to the sugar and to the odor should be visible in a test phase, thus confirming the aversive nature of the substances used to devaluate the reward.

The experiment consisted of three consecutive phases: 1) CS-US association phase, 2) US devaluation phase, and 3) Test of US and CS responsiveness.

### CS-US association phase

We harnessed honeybees in individual metal holders and after two and half hours of rest, we first measured US responsiveness by touching the antennae with water (control) and then, 15 min later, with the US. Three different sugars, all at a concentration of 30% (weight/weight) were used as US, each with a different group of bees: fructose 1.66 M (fructose group), glucose 1.66 M (glucose group) and sucrose 1 M (sucrose group). These sugars have different nutritional values and differ, therefore, in their attractiveness for free-flying honeybees in the field and freely-moving bees in the laboratory [43]. Based on previous results [40], the ranking expected was fructose < glucose < sucrose. In all cases, we recorded whether or not bees extended the proboscis to the stimulus tested. We report for each case the % of PER elicited by each stimulus. One-factorial and repeated measurement anova was used for comparisons between and within groups, respectively.

Bees of the three groups (fructose, glucose and sucrose) were then conditioned with four pairings of 1-nonanol (CS) and their respective US. Conditioning trials followed the same dynamics as in Experiment 1 (see above) and were separated by 14 min. We quantified whether or not bees extended the proboscis to the conditioned odor 1-nonanol during the four conditioning trials. Variations in acquisition within and between groups were analyzed using ANOVA for repeated measurements [38].

### US devaluation phase

After the last conditioning trial of the CS-US association phase, bees experienced a 40 min rest. Afterwards, they were fed during 50 min with 20 µl (4×5 µl, as in Experiment 2) of an aversive compound or water (control) that could be paired or not with the US delivered to the antennae (fructose, glucose or sucrose depending on the group). Thus, harnessed bees of the ***paired group*** experienced four trials in which they received stimulations of the US on the antennae, which elicited PER and which were followed by delivery of an aversive compound or water to the proboscis. Adjacent trials were separated by 12 to13 min. The temporal dynamics of each trial was similar to that of the CS-US association phase except that the odor (CS) stimulation was replaced by the antennal stimulation with the US (fructose, glucose or sucrose depending on the group) and that US feeding was replaced by feeding an aversive substance or water to the proboscis. Harnessed bees of the ***unpaired group*** experienced the same stimulations (US on the antennae and an aversive solution or water to the proboscis) but in a non-contingent way so that association between the US and the aversive compound was excluded. Adjacent trials were spaced by 12–13 min.

We reasoned that if the contact with aversive substances generates a distasteful sensory experience, pairing the US with them would lead to reward devaluation, and thus to CS devaluation. On the contrary, the unpaired group would not exhibit such devaluation due to the lack of contingency between the US and the distasteful experience generated by the aversive substances. If, on the other hand, these substances are acceptable in terms of their taste but once ingested they generate a posterior malaise, pairing them or not with the US would have the same effect: malaise would follow ingestion in both cases so that a decrease in US and CS responses would be evident in both paired and unpaired groups.

Bees of the unpaired group needed to be placed in the conditioning setup 8 times (4 times for antennal stimulation with sugar only, and 4 times for feeding at the level of the proboscis; trials followed a pseudorandom sequence). Thus, bees of the paired group had to experience 4 blank trials interspersed in a pseudorandom way with associative trials in order to equate the number of placements in the conditioned setup (8) between paired and unpaired groups.

The solutions fed to the bees in the 2^nd^ phase were distilled water (control), quinine 10 mM, LiCl 140 mM and amygdalin 1 M. These substances were chosen based on the results of Experiment 2 (2^nd^ experimental series) as they do not induce highest mortality but affect nevertheless the probability of survival. Thus, for each group (fructose, glucose and sucrose), there were four subgroups (water, quinine, LiCl and amygdalin) each of which was subdivided in paired and unpaired subgroups. Twenty four groups of bees were therefore studied in this experiment.

After the last feeding to the proboscis, a resting period of 90 min was introduced before performing CS and US tests. This period was chosen based on the results of Experiment 2 and corresponded to a decrease in the probability of survival of only 20%. In this way, malaise, if any, should already exert an action in the bees treated having ingested the 20 µl of aversive substances but should not lead to a significant bee loss, which would impede completing the last phase of the experiment.

### Test of CS and US responsiveness

After the 90 min rest, we tested the response of bees of the different groups to their respective US (fructose, glucose or sucrose) and to the CS, 1-nonanol, in absence of US. US responsiveness was assessed by touching the antennae with the corresponding US (sucrose, glucose or fructose). To determine the extent to what the CS response was specific, we also tested bees with a novel odorant, which was not used during conditioning. In order to avoid odor generalization, which plays an important role in odor responses in bees [44], we chose as novel odorant 1-hexanol which is well differentiated from 1-nonanol [45]. Odors were given in a random sequence, which varied from bee to bee. Within each group, we compared responses to the CS and to the novel odor using McNemar's test. Comparisons between groups were done by means of a χ^2^ test.

## Results

### Experiment 1

In the pre-exposure phase, we trained bees to associate 1-nonanol with different non-appetitive reinforcements, including deterrent substances; in a 2^nd^ conditioning phase, we trained the same bees to associate 1-nonanol with sucrose 1 M. We aimed at determining whether or not deterrent substances hinder appetitive acquisition of 1-nonanol in the 2^nd^ phase due to an aversive associative strength gained in the pre-exposure phase.

**Figure 1:**
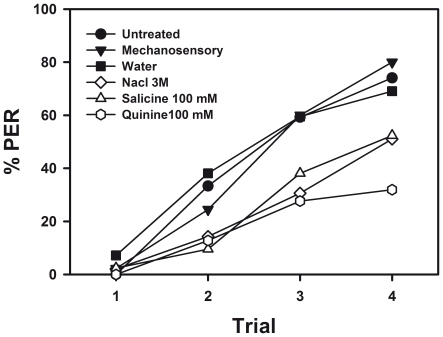
Effect of pre-exposure to aversive substances on olfactory appetitive learning in harnessed honeybees. The graph shows the performance (percentage of proboscis extension responses or PER) of honeybees during four trials of appetitive olfactory conditioning in which the odor 1-nonanol was paired with sucrose 1 M. Prior to this conditioning phase, bees were pre-exposed to 1-nonanol^a^ paired either with a mechanosensory stimulus (n = 45), distilled water (n = 42), NaCl 3 M (n = 49), salicine 100 mM (n = 42) or quinine 100 mM (n = 47). The untreated^b^ group (n = 54) was not pre-exposed. Bees having experienced NaCl, salicine and quinine showed lower acquisition than the other groups (water, mechanosensory and untreated)^c^. No significant differences in acquisition were found between bees of the untreated, mechanosensory and water group. Different letters indicate significant between-group differences.

In the first pre-exposure phase, bees of the five groups (quinine 100 mM: n = 47; salicine 100 mM: n = 42; NaCl 3 M: n = 49; water: n = 42, and mechanosensory: n = 45) received four pairings of 1-nonanol with their respective reinforcement. One group was left untreated (n = 54). In total, 279 bees were used in this experiment. None of the treated groups exhibited conditioned responses to 1-nonanol during the four conditioning trials (not shown). Thus, no treatment, be it quinine, salicine, NaCl, water, or mechanosensory stimulation, supported appetitive learning of 1-nonanol. The analysis of group performances focused therefore on the responses during the 2^nd^ phase, in which all six groups received four pairings of 1-nonanol with sucrose solution 1 M (Fig. 1).

All six groups exhibited a significant acquisition of the odor-sucrose association in the conditioning phase (Fig. 1; *untreated*: *F*_3,159_ = 50.59, *p*<0.00001; *water*: *F*_3,123_ = 26.38, *p*<0.00001; *mechanosensory*: *F*_3,132_ = 47.37, *p*<0.00001; *NaCl*: *F*_3,144_ = 19.98, *p*<0.00001; *quinine* 100 mM: *F*_3,138_ = 12.55, *p*<0.00001; *salicine*: *F*_3,123_ = 20.22, *p*<0.00001). However, acquisition levels significantly differed between groups (*F*_5,273_ = 7.28, *p*<0.00001) and the interaction group x trial was also significant (*F*_15,819_ = 3.16, *p*<0.00005). Clearly, untreated bees, which did not experience any treatment in the pre-exposure phase, did not differ in their acquisition performance from bees that received a neutral stimulus such as a mechanosensory stimulation or water (*F*_2,138_ = 0.06, *NS*). On the contrary, bees that ingested quinine 100 mM, salicine 100 mM or NaCl 3 M in the pre-exposure phase exhibited a significantly lower acquisition than the control group that ingested water (*quinine vs. water*: *F*_1,87_ = 16.08, *p*<0.0005; *salicine vs. water*: *F*_1,82_ = 7.91, *p*<0.01; *NaCl vs. water*: *F*_1,89_ = 9.41, *p*<0.005). Although a comparison between these three groups was not significant (*F*_2,135_ = 1.06, *NS*), the quinine group was the only one that exhibited a significant interaction group x trial when compared to the water control (*F*_3,261_ = 3.84, *p*<0.02), thus indicating a different variation of conditioned responses during trials. Figure 1 shows indeed that at the end of acquisition the percentage of conditioned responses was lower in the group that ingested quinine 100 mM during the pre-exposure phase (*p*<0.05). Thus, from the three substances that induced a retardation of acquisition, NaCl 3 M, salicine 100 mM and quinine 100 mM, quinine seems to have a more drastic effect visible at the end of conditioning.

The question arises as to which pathway was involved in the retardation effect induced by NaCl, salicine and quinine. On one hand, retardation could be due to a distasteful sensory experience generated by the contact with these solutions. On the other hand, these substances could be acceptable in terms of their taste but once ingested they could generate a posterior malaise responsible for the retardation of acquisition. In order to evaluate these possibilities, we performed an experiment in which we studied whether bees ingest or not considerable quantities of these and other aversive substances and quantified post-ingestional mortality.

### Experiment 2

In both experimental series, bees ingested the control solution (distilled water) without reluctance. Surprisingly, they also did it for all aversive substances used so that at the end of the feeding, all harnessed bees had consumed the 20 µl of aversive substances, irrespective of their taste. No obvious differences were seen in terms of feeding behavior between bees ingesting water or aversive compounds.

**Figure 2:**
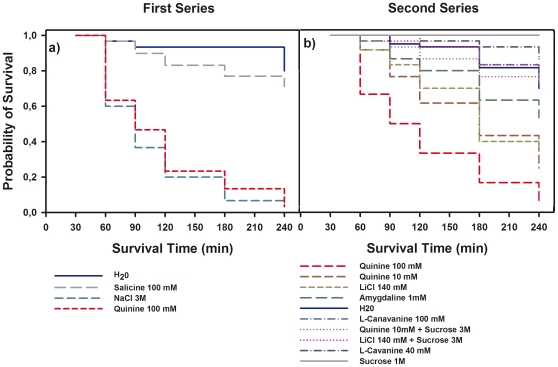
Kaplan–Meier curves of survival for harnessed honeybees following feeding of aversive compounds. (**a**) ***First series***. The probability of survival differed significantly between groups (long-rank test: *χ^2^* = 64.07, df:3, *p*<0.0001). The group of honeybees having ingested NaCl 3 M (n = 30) and quinine 100 mM (n = 30) exhibited a significant decrease of their survival probability compared to the distilled water group (n = 30). The group having ingested salicine 100 mM (n = 30) had intermediate mortality levels and comparison to the distilled water group, which exhibited a low decrease of the probability of survival, was marginally non-significant (*Z* = 1.78, *p* = 0.07). (**b**) ***Second series***. The probability of survival differed significantly between groups (long-rank test: *χ^2^* = 108.93, df:8, *p*<0.0001). The group of bees having ingested sucrose 1 M group (n = 30) did not exhibit any variation of their probability of survival over time. The quinine 100 mM group (n = 30) experienced higher mortality than the distilled water group (n = 60). The quinine 10 mM (n = 60) and LiCl 140 mM (n = 60) groups experienced also induced higher mortality than the distilled water group. The amygdaline 1 mM group (n = 30) exhibited inetrmediate mortality compared to the the distilled water group. Mortality in the L-canavanine 40 mM (n = 30) and 100 mM (n = 30) groups was not significantly different from that of the distilled water group. The probability of survival from the groups having ingested mixtures of quinine 10 mM and sucrose 1 M (n = 30) and LiCL 140 mM and sucrose 1 M (n = 30) did not differ from that of the distilled water group.

In a *first series*, we used the solutions that induced a retardation of acquisition in Experiment 1: NaCl 3 M (n = 30), salicine 100 mM (n = 30), quinine 100 mM (n = 30), and distilled water (n = 30) as a control. In total 120 bees were used in this experimental series. Figure 2a shows that despite the fact that bees ingested all four solutions without reluctance, the probability of survival differed significantly between groups (log-rank test: *χ^2^* = 64.07, df:3, *p*<0.0001). Bees having ingested NaCl 3 M and quinine 100 mM exhibited highest and similar mortality levels (*Z* = 0.63, *NS*) so that their probability of survival decreased dramatically following ingestion; feeding of water induced only a slight but not significant increase of mortality at the end of the experiment probably due to exhaustion (240 min); salicine 100 mM induced intermediate mortality levels and comparison to the water group was marginally non-significant (*Z* = 1.78, *p* = 0.07). These results suggest that although quinine 100 mM, NaCl 3 M and salicine 100 mM induced a retardation of acquisition in Experiment 1, their aversive effects were probably due to different processes. While a concentrated NaCl solution disrupts osmotic equilibrium and leads to death, concentrated quinine solution seems to induce a post-ingestional malaise-like state that also determines high mortality. Salicine, on the other hand, induced lower mortality despite its high concentration, thus suggesting that its aversive effect was due to a gustatory deterrent effect rather than to a malaise-like state.

In the *second series*, we expanded the spectrum of deterrent substances fed to the bees. Besides a solution of quinine 100 mM (n = 30) and a control of distilled water (n = 60) also used in the first series, we included a diluted quinine solution (10 mM; n = 60), a solution of LiCl 140 mM (n = 60), a solution of amygdalin 1 mM (n = 30), two concentrations of L-canavanine (40 and 100 mM; n = 30 in both cases), a sucrose solution 1 M (n = 30), and mixtures of sucrose 1 M and Licl 140 mM (n = 30) and of sucrose 1 M and quinine 10 mM (n = 30). In total 390 bees were used in this experimental series.

Figure 2b shows that the solutions fed induced different mortality levels, thus determining different probabilities of survival (log-rank test: *χ^2^* = 108.93, df:8, *p*<0.0001). While feeding of sucrose 1 M did not induce any mortality over time (and was therefore excluded from analyses as it only included censored data), feeding of water induced only a slight increase of mortality at the end of the experiment probably due to exhaustion (180 and 240 min). Feeding of quinine, on the other hand, induced highest levels of mortality as in the first series. When compared to the water control, mortality induced by quinine was highly significant (quinine 100 mM: *Z* = 6.37, *p*<0.001; quinine 10 mM: *Z* = 5.34, *p*<0.001). Significant differences were found between the two concentrations of quinine used (*Z* = 2.95, *p*<0.005), thus showing a dose-dependent effect for this substance. Indeed, the quinine 100 mM group reached 50% mortality at approximately 90 min post ingestion, while the quinine 10 mM group did it between 120 and 180 min post ingestion. Higher post-ingestional mortality levels were also found for LiCl 140 mM. In this case, survival probability was significantly different from that of the water control (*Z* = 4.36, *p*<0.001) and from that of the quinine 100 mM group (*Z* = 3.50, *p*<0.0005), but did not differ from that of the quinine 10 mM group (*Z* = 0.90, *NS*). Thus, after ingestion of LiCl 140 mM, 50% mortality was reached between 120 and 180 min, as for the quinine 10 mM group.

Besides quinine and LiCl, ingestion of amygdalin 1 mM also reduced the probability of survival in a significant way when compared to the water control (*Z* = 2.80, *p*<0.01). In this case, probability of survival did not differ from that of the quinine 10 mM group (*Z* = 1.22, *NS*) but was significantly higher than that of the group quinine 100 mM (*Z* = 3.61, *p*<0.001). L-cavanine, both at a low (40 mM) and high concentration (100 mM), had no effect as in both cases mortality did not differ from that induced by the water control (L-cavanine 40 mM: *Z* = 1.59, *NS*; L-canavanine 100 mM: *Z* = 0.19; *NS*). No differences were found between these two concentrations (*Z* = 1.28, *NS*). Finally, despite a slight increase of mortality visible in the latest measurement times (180 and 240 min), no significant differences were found between the probability of survival following ingestion of the mixtures of quinine 10 mM and sucrose 1 M and LiCl 140 mM and sucrose 1 M (*Z* = 0.38, *NS*). Interestingly, the probability of survival induced by the mixtures did not differ from that associated with water ingestion (quinine 10 mM + sucrose 1 M: *Z* = 0.0004, *NS*; LiCl 140 mM + sucrose 1 M: *Z* = 0.46, *NS*), thus meaning that the same substances, quinine 10 mM and LiCl 140 mM, that induced important and comparable levels of mortality when ingested alone, lose their toxic effect when mixed with sucrose solution 1 M, which yields full survival when ingested alone. All in all, the results of the *second series* show again that harnessed bees drink deterrent substances such as LiCl, quinine, amygdalin and L-canavanine and that ingestion of quinine, LiCl, and amygdalin yields significant mortality, probably because of a toxic, post-ingestional effect of these substances.

### Experiment 3

This experiment consisted of three consecutive phases: 1) CS-US association phase, 2) US devaluation phase with paired (US contingent to the ingestion of aversive compounds) and unpaired groups (US non contingent to the ingestion of aversive compounds), and 3) Test of US and CS responsiveness in both the paired and the unpaired group. We analyzed whether ingestion of aversive compounds in the 2^nd^ phase induces US and CS devaluation in the paired group, consistent with a distasteful gustatory experience, or whether devaluation is common to both the paired and the unpaired group, consistent with a non-specific malaise induced by the ingestion of aversive compounds.

**Figure 3:**
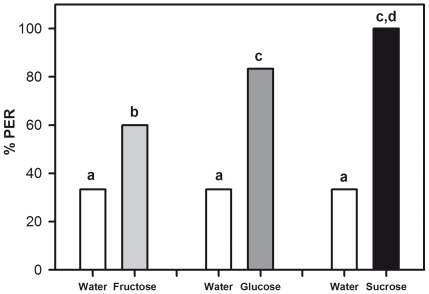
Ranking of sugar solutions by harnessed bees. The graph shows the percentage of proboscis extension responses (PER) upon antennal stimulation with fructose 1,66 M, glucose 1,66 M and sucrose 1 M. Each sugar was assayed with a different group of bees experiencing also a control stimulation with distilled water control (n = 30 each). Bees responded significantly more to the sugar than to the water. The preference ranking was fructose < glucose < sucrose. Different letters indicate significant between-group differences.

Three different sugars, all at a concentration of 30% (weight/weight) were used as US: fructose 1.66 M (fructose group), glucose 1.66 M (glucose group) and sucrose 1 M (sucrose group). We first tested US responsiveness by touching the antennae with these sugars and measuring PER. Figure 3 shows the % of PER of three different groups of bees (n = 30 each) to these substances, together with their respective water control. An Anova for Repeated Measurements showed that in all three groups, bees responded significantly more to the sugar than to the water (*F*_1,87_ = 89.22, *p*<0.0001). Moreover, the interaction was significant, thus showing that the pattern of responses varied depending on the sugar tested (*F*_2,87_ = 5.26, *p*<0.01). Specifically, while responses to water were constant and remained at a 33% level (*F*_2,87_ = 0, *NS*), responses to sugar significantly increased from fructose to sucrose (*F*_2,87_ = 9.27, *p*<0.001), so that sugars were ranked as follow: fructose < glucose < sucrose (post hoc Fisher test: *p*<0.05).

### Fructose as US

**Figure 4:**
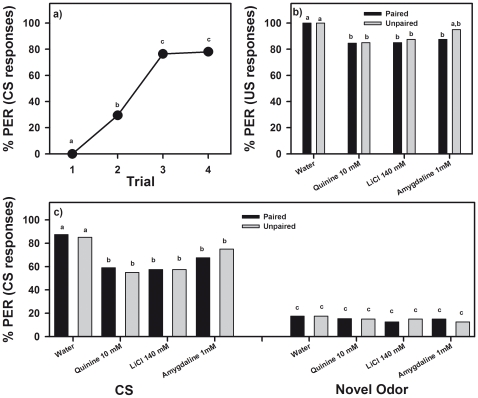
Devaluation of fructose 1.66 M. The graph shows the performance (percentage of proboscis extension responses or PER) during (**a**) an odor-fructose association in which the response to the odor (conditioned stimulus or CS) was quantified, and during (**b,c**) a test phase following a devaluation phase in which responses to the sugar (US; see **b**) and to the odor (CS see **c**) were quantified in paired and unpaired groups of bees experiencing or not an association between sugar and either distilled water, quinine 10 mM, LiCl 140 mM or amygdaline 1 mM (319 bees in total). (**a**) All bees learned the odor-fructose association. The graph shows the pooled acquisition performance of all eight groups of bees. (**b**) Ingestion of quinine, LiCl and amygdaline decreased US responsiveness with respect to a water control. Responses of paired and unpaired groups were similar. (**c**) Ingestion of quinine, LiCl and amygdaline decreased CS responsiveness with respect of a water control. Responses to a novel odor remained low and equivalent in all groups. Different letters indicate significant between-group differences.

Eight groups of bees (319 bees in total) were subjected to a four-trial conditioning with 1-nonanol as CS and fructose 1.66 M as US. All groups learned similarly to respond to the rewarded odor and increased proboscis extension responses to the odor during trials (group effect: *F*_7,933_ = 0.07, *NS*; trial effect: *F*_3,933_ = 481.15, *p*<0.0001). The interaction group x trial was not significant, thus showing that all groups exhibited the same learning dynamic along trials (*F*_21,933_ = 0.11, *NS*). A single curve is therefore presented in Fig. 4a showing the pooled acquisition performance of all eight groups of bees.

After acquisition, the eight groups were subjected to different treatments in a US devaluation phase. Four groups received antennal fructose stimulation paired with ingestion of either distilled water (control), quinine 10 mM, LiCl 140 mM or amygdalin 1 mM. The other four groups received the same stimulations but these were unpaired as to exclude associative effects. Figure 4b shows the response to the US of paired and unpaired groups after the devaluation phase. As each bar corresponds to a different group, a one-factor anova was used to detect differences in US responses. Bees responded differently to the US (fructose 1.66 M), depending on the treatment experienced in the US devaluation phase (*F*_7,311_ = 2.15, *p*<0.05). Fisher post hoc tests revealed that the control treatment (distilled water, either paired or unpaired) did not modify US responsiveness, which stayed at a 100% level; however ingestion of quinine 10 mM, LiCl 140 mM and to a lesser extent of amygdalin 1 mM decreased significantly US responsiveness (Fig. 4b; Fisher post hoc tests, *p*<0.05), thus revealing a US devaluation effect. Notably, this effect was common to both paired and unpaired groups (*F*_1,317_ = 0.61, *NS*), which indicates that rather than being due to an associative link between US and aversive taste, US devaluation was due to a generalized malaise induced by the ingestion of aversive compounds, which affected in a non-specific way US responsiveness.

Such an effect was also reflected in CS responsiveness (Fig. 4c). Bees presented with 1-nonanol, the CS, and with a novel odor, 1-hexanol, responded significantly to the CS and not to the novel odor (*F*_1,311_ = 350.32, *p*<0.0001), and although differences between treatments were marginally non-significant (*F*_7,311_ = 1.91, *p* = 0.06), the interaction odor x treatment was significant (*F*_7,311_ = 2.28, *p*<0.05) thus showing that odor responses varied depending on the treatment experienced in the US devaluation phase. Fisher post hoc tests revealed that the control treatment (distilled water, either paired or unpaired) did not modify CS responsiveness, which stayed at a 85% level. However, ingestion of quinine 10 mM, LiCl 140 mM and amygdalin 1 mM decreased significantly CS responsiveness (Fig. 4c; Fisher post hoc tests, *p*<0.05). The responses to the novel odor remained low in all cases, both for the paired and the unpaired treatments (Fig. 4c). These results show that besides the US devaluation effect, a CS devaluation effect was induced by these substances. Again, this effect was common to both paired and unpaired groups (*F*_1,317_ = 0.0002, *NS*), which indicates that rather than being due to an associative link between US and aversive taste, CS devaluation was due to a generalized malaise induced by the ingestion of aversive compounds, which affected in a non-specific way CS responsiveness.

### Glucose as US

**Figure 5:**
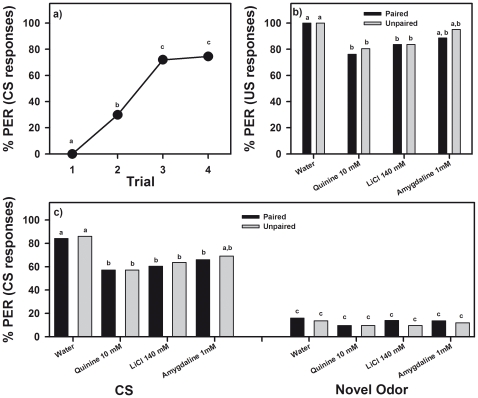
Devaluation of glucose 1.66 M. The graph shows the performance (percentage of proboscis extension responses or PER) during (**a**) an odor-glucose association in which the response to the odor (conditioned stimulus or CS) was quantified, and during (**b,c**) a test phase following a devaluation phase in which responses to the sugar (US; see **b**) and to the odor (CS see **c**) were quantified in paired and unpaired groups of bees experiencing or not an association between sugar and either distilled water, quinine 10 mM, LiCl 140 mM or amygdaline 1 mM (319 bees in total). (**a**) All bees learned the odor-glucose association. The graph shows the pooled acquisition performance of all eight groups of bees. (**b**) Ingestion of quinine, LiCl and amygdaline decreased US responsiveness with respect to a water control. Responses of paired and unpaired groups were similar. (**c**) Ingestion of quinine, LiCl and amygdaline decreased CS responsiveness with respect of a water control. Responses to a novel odor remained low and equivalent in all groups. Different letters indicate significant between-group differences.

Eight groups of bees (342 bees in total) were subjected to a four-trial conditioning with 1-nonanol as CS and glucose 1.66 M as US. All groups learned similarly to respond to the rewarded odor and increased proboscis extension responses to the odor during trials (group effect: *F*_7,1002_ = 0.08, *NS*; trial effect: *F*_3,1002_ = 456.38, *p*<0.0001). As for fructose, the interaction group x trial was not significant, thus showing that all groups exhibited the same learning dynamic along trials (*F*_21,1002_ = 0.19, *NS*). A single curve is therefore presented in Fig. 5a showing the pooled acquisition performance of all eight groups of bees.

After acquisition, the eight groups were subjected to different treatments in a US devaluation phase. Four groups received antennal glucose stimulation paired with ingestion of either distilled water (control), quinine 10 mM, LiCl 140 mM or amygdalin 1 mM. The other four groups received the same stimulations but these were unpaired as to exclude associative effects. Figure 5b shows the response to the US of paired and unpaired groups after the devaluation phase. Bees responded differently to the US of glucose 1.66 M, depending on the treatment experienced in the US devaluation phase (*F*_7,334_ = 3.62, *p*<0.001). Fisher post hoc tests showed that the control treatment (distilled water, either paired or unpaired) did not modify US responsiveness, which stayed at a 100% level; however ingestion of quinine 10 mM, LiCl 140 mM and to a lesser extent of amygdalin 1 mM decreased significantly US responsiveness (Fig. 5b; Fisher post hoc tests, *p*<*0.05*), thus revealing a US devaluation effect. As in the case of fructose, this effect was common to both paired and unpaired groups (*F*_1,340_ = 0.60, *NS*), which indicates that US devaluation was not due to an associative link between US and aversive taste, but rather to a generalized malaise induced by the ingestion of aversive compounds.

Bees presented with 1-nonanol, the CS, and with a novel odor, 1-hexanol, responded significantly to the CS and not to the novel odor (Fig. 5c; *F*_1,334_ = 375.42, *p*<0.0001). Differences between treatments were marginally non-significant (*F*_7,334_ = 1.77, *p* = 0.09) and the interaction odor x treatment was not significant (*F*_7,334_ = 1.44, *NS*). However, Fisher post hoc tests showed that there were significant differences between groups consistent with a CS devaluation effect induced by the ingestion of aversive compounds. While the control treatment (distilled water, either paired or unpaired) did not modify CS responsiveness, which stayed at a 85% level, quinine 10 mM, LiCl 140 mM and amygdalin 1 mM decreased it significantly (Fig. 5c; Fisher post hoc tests, *p*<0.05). In all cases, responses to the novel odor remained low (Fig. 5c). Thus, a CS devaluation effect, common to both paired and unpaired groups (*F*_1,340_ = 0.22, *NS*), was induced by aversive compounds. This result again indicates that CS devaluation was due to a generalized malaise induced by the ingestion of aversive compounds.

### Sucrose as US

**Figure 6:**
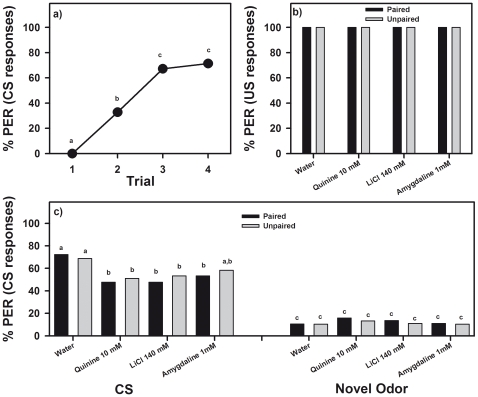
Devaluation of sucrose 1 M. The graph shows the performance (percentage of proboscis extension responses or PER) during (**a**) an odor-sucrose association in which the response to the odor (conditioned stimulus or CS) was quantified, and during (**b,c**) a test phase following a devaluation phase in which responses to the sugar (US; see **b**) and to the odor (CS see **c**) were quantified in paired and unpaired groups of bees experiencing or not an association between sugar and either distilled water, quinine 10 mM, LiCl 140 mM or amygdaline 1 mM (319 bees in total). (**a**) All bees learned the odor-fructose association. The graph shows the pooled acquisition performance of all eight groups of bees. (**b**) Ingestion of quinine, LiCl and amygdaline did not diminish US responsiveness with respect to a water control. Bees responded maximally to sucrose (100% PER). Responses of paired and unpaired groups were similar. (**c**) Ingestion of quinine, LiCl and amygdaline decreased CS responsiveness with respect of a water control. Responses to a novel odor remained low and equivalent in all groups. Different letters indicate significant between-group differences.

As in the previous series, eight groups of bees (366 bees in total) were subjected to a four-trial conditioning with 1-nonanol as CS and sucrose 1 M as US. All groups learned similarly to respond to the rewarded odor and increased proboscis extension responses to the odor during trials (group effect: *F*_7,1074_ = 0.08, *NS*; trial effect: *F*_3,1074_ = 403.59, *p*<0.0001; interaction group x trial: *F*_21,1074_ = 0.18, *NS*). A single curve is therefore presented in Fig. 6a showing the pooled acquisition performance of all eight groups of bees.

After acquisition, the eight groups were subjected to a US devaluation phase. Four groups received antennal sucrose stimulation paired with ingestion of distilled water (control), quinine 10 mM, LiCl 140 mM or amygdalin 1 mM. The other four groups received the same stimulations but these were unpaired as to exclude associative effects. Figure 6b shows the response to the US of paired and unpaired groups after the devaluation phase. In all case, bees responded maximally to sucrose (100% PER) so that there were no differences between groups and no devaluation effect was evident (Fig. 6b). This absence of US devaluation was common to both paired and unpaired groups, which indicates that sucrose 1 M is a particularly powerful US, capable of overcoming the malaise induced by aversive compounds.

Bees presented with 1-nonanol, the CS, and with a novel odor, 1-hexanol, responded significantly to the CS and not to the novel odor (Fig. 6c; *F*_1,358_ = 245.26, *p*<0.0001). No significant differences were found between treatments (*F*_7,358_ = 0.67, *NS*). However, the interaction odor x treatment was marginally non-significant (*F*_7,358_ = 1.98, *p* = 0.06). Fisher post hoc tests showed that there were significant differences between groups consistent with a CS devaluation effect induced by the ingestion of aversive compounds. As in the previous series (glucose and fructose as US), the control treatment (distilled water, either paired or unpaired) did not modify CS responsiveness, which stayed at a 70% level, quinine 10 mM, LiCl 140 mM and amygdalin 1 mM decreased it significantly (Fig. 6c; Fisher post hoc tests, *p*<0.05). Responses to the novel odor remained low (Fig. 6c). Thus, a CS devaluation effect, common to both paired and unpaired groups (*F*_1,364_ = 0.005, *NS*), was induced by aversive compounds. This result shows that ingestion of aversive compounds induced CS devaluation and that US devaluation was not apparent because sucrose 1 M was able to overcome it.

## Discussion

The present work shows that despite their distasteful nature, aversive compounds are ingested by harnessed bees when offered in high concentrations, and that they exert, in most of the cases, a post-ingestional malaise. Our results indicate, therefore, that substances that taste bitter to humans such as quinine, salicine, and amygdalin, as well as concentrated saline solutions (NaCl, LiCl) base their aversive effect on the physiological consequences that their ingestion generates in harnessed bees rather than on an unpalatable taste. Such effect would lead to immediate rejection of these substances upon contact with the gustatory organs (antennae, proboscis), a response that was never observed in all the experiments performed.

### Distasteful gustatory experiences vs. non-specific malaise induced by aversive compound ingestion

In all cases, harnessed bees ingested considerable amounts of aversive substances. Twenty µl were ingested without any visible reluctance or rejection in Experiments 2 and 3. This applied to substances that may taste very different to bees such as concentrated saline solutions (NaCl or LiCl) or substances that are bitter for humans. Twenty µl, which represent one third of the honeybee crop [32], [46], constitute the limit that was defined by the experimenter, not by the bee. It is, therefore, possible that in our experimental conditions, harnessed bees would ingest even higher volumes of the aversive compounds assayed. This result excludes *a priori* the fact that bees in our experimental conditions experienced distasteful gustatory sensations upon contact with the solutions used in our experiments. Should they have experienced such sensations, then they would have expressed a clear rejection behavior. This was never the case.

The lack of aversion evinced in our experiments is in agreement with previous findings obtained in the same experimental conditions (harnessed bees in the laboratory) showing that bees do not exhibit significant rejection behavior when stimulated with substances like quinine or salicine [6]. They also coincide with the fact that until now, no specific receptor neuron tuned to these aversive substances has been found in electrophysiological experiments performed at the level of the honeybee antennae, where most gustatory receptor classes (responding to sucrose and saline solutions) are grouped [6]. Moreover, no equivalent of the fruit fly receptor genes Dm Gr66a and DmGr93a, tuned to respond to bitter compounds [12]–[14], could be identified in the honeybee genome [20].

Note, however, that gustatory receptor neurons responding to substances like quinine or salicine may be located in sensillae of difficult access and may have remained undetected until now by electrophysiological recordings. In the same way, the absence of homologies between the ‘aversive’ Grs of the fruit fly and the reduced number of honeybee Grs (only 10) [20] is not a conclusive evidence for the absence of ‘bitter’ taste in bees. While *Drosophila* Grs encode putative heptahelical 7-transmembrane proteins, it is not clear whether the resulting gustatory receptors signal through G-protein-dependent 2^nd^ messenger cascades or operate as ligand-gated ion channels. Recently, DmX, a gustatory receptor of the fruit fly tuned to detect the natural toxic molecule L-canavanine used in our work has been explicitly identified as a G-protein-coupled receptor [40]. Interestingly, this DmX receptor has partially diverged in its ligand binding pocket from the metabotropic glutamate receptor family and is not related to the Gr family. The expression of the DmX receptor is required in bitter-sensitive gustatory receptor neurons (i.e. in neurons expressing Dm Gr66a), where it triggers the premature retraction of the proboscis of the fly, thus leading to the end of food searching and food aversion [40]. In our experiments, L-canavanine had no significant effect on honeybee mortality. Similarly, fruit flies that eat this substance suffer no dramatic effects themselves, but all of their offspring die as larvae [40]. Honeybee workers are sterile and have no offspring so that a comparable effect could be studied by checking the effect of L-canavanine ingestion on the offspring of honeybee queens.

Although we do not exclude the existence of ‘bitter’ taste sensations in bees, in the experimental conditions of the present work, these were not apparent. Only Experiments 1 and 2 suggested that salicine, which like quinine solution induced retardation of acquisition in Experiment 1 but which contrarily to quinine did not induce significant mortality levels despite the high concentration used, may produce an aversive gustatory experience, responsible of retardation in Experiment1. However, the effect of the great majority of substances tested was not explainable in terms of their aversive taste because bees consumed them without evident rejection. On the contrary, the notion that ingestion of a considerable amount of these substances led to a post-ingestional malaise-like state in the case of some of these substances is supported by the levels of resulting mortality found in Experiment 2 and by the generalized devaluation of US and CS common to paired and unpaired groups in Experiment 3.

Post-ingestional malaise caused by the injection of the toxin nicotine hydrogen tartrate has been shown in the grasshopper *Schistocerca Americana* [47]. This insect is capable of learning to associate the gustatory cues of an initially acceptable novel food with post-ingestional malaise caused by this substance. The learned association was manifested in avoidance or reduction in acceptability of food [47]. In further experiments [48], grasshoppers were injected with ouabain, a cardiac glycoside, coumarin, a lactone, quinine hydrochloride and LiCl. All substances induced post-ingestional malaise leading to a reduction of food acceptability and retention of learned aversion lasted at least two days [48]. It was argued that these capacities are adaptive in an ecological context because learning to associate the sensory cues of a food plant with its post-ingestional consequences could allow a polyphagous herbivore like the grasshopper, which may contact a wide variety of plants differing in the toxins they contain, to avoid or limit the ingestion of toxic foodplants [48].

In our case, similar arguments could apply to the ingestion of toxic nectars by honeybees. Although we chose the oral way to deliver the aversive substances rather than injecting them, which could produce uncontrolled damages in a small insect like the honeybee, the malaise generated by substances like LiCl, quinine and amygdalin (Experiment 3) led the bees to decrease their responses to an odorant that was previously appetitive. The effect was present both in the paired and the unpaired group thus suggesting a generalized malaise independently of the temporal association between the sugar and the aversive compound in the devaluation phase. This may be due to the fact that it is not the explicit US-aversive compound pairing (or non-pairing) that is learned given that no aversive taste experience is involved but rather the fact that US delivery was followed by a malaise that developed later, after paired or non-paired presentations have finished. Experiments on conditioned taste aversion (CTA), a form of learning which develops when a novel taste is associated with a short-term unpleasant gastrointestinal sensation [49], [50], have clearly shown that rodents associate gustatory and olfactory cues with internal malaise even when these stimuli are separated by long periods of time [51]. This kind of learning, also evident in the devaluation experiments reported in Experiment 3, show that animals – and bees are not an exception – possess an inherent ability to selectively associate gustatory cues with nausea or malaise and that this associative mechanism is mediated by a system that enables the association to form over extended delays. As argued by Freeman and Riley [52], this particular feature of CTA makes sense in an ecological context and helps individual survival given that toxicity is likely to follow consumption of a toxin after some delay.

### Mechanisms of malaise-like state in honeybees

In analyzing this post-ingestional malaise, it is possible to discern different main sources of physiological distress that could explain retardation of acquisition (Experiment 1), the increased levels of mortality following ingestion (Experiment 2) and US and CS devaluation (Experiment 3) following ingestion of some aversive substances. On one hand, concentrated saline solutions may have disrupted osmotic equilibrium thus leading to death. Sodium and chloride are primary electrolytes involved in cellular osmosis; in vertebrates, their excessive consumption can lead to muscle cramps, dizziness, or electrolyte disturbance, which can cause neurological problems, or be fatal. Thus, low probability of survival following NaCl ingestion (see Experiment 2) can be related to similar physiological consequences. LiCl, on the other hand, is commonly used in experiments on conditioned taste aversion (CTA) in vertebrates and is particularly effective in generating CTA [50], [53]. LiCl administration, usually via peritoneal injection, causes a significant reduction in food intake, gastric motility, and gastric emptying [54], [55]. Peripheral injection of LiCl results in the onset of complex neural and endocrine mechanisms that underlie the development of anorexic and aversive responses in rodents [56]. Although the mechanisms by which LiCl acts on honeybees are unknown, it is clear that, as in vertebrates, it has a toxic effect that induces significant mortality between 2 and 3 h after ingestion.

Substances like quinine and amygdalin, whose ingestion significantly affected the probability of survival, may induce malaise through different mechanisms. Quinine is a quinoline alkaloid that has local anesthetic action but is also a local irritant of the gut in humans. The irritant effects may be responsible in part for the nausea associated with the clinical use of quinine [57]. To our knowledge, the mechanisms by which quinine can induce nausea-like sensations in insects are unknown but if higher doses of quinine exert similar irritant properties on the insect gut, the malaise evinced in our work could be understood. Amygdalin, on the other hand, is a cyanogenic glycoside whose toxicity for humans is related to the presence of an enzyme in the small intestine – the beta glucosidase – that catalyzes the release of the cyanide from amygdalin. This release may lead to lethal toxicity upon oral ingestion of amygdalin [58]. Beta glucosidase has also been purified from the ventriculus and honey sac of *Apis mellifera* [59] where it seems to be related to pollen digestion. It could have similar consequences as in humans (increased cyanide levels in hemolymph), thus accounting for the toxicity of this substance when higher doses are consumed by the bees.

Mixing LiCl and quinine with sucrose solution suppressed the toxicity of these two aversive compounds. As shown by Experiment 3, sucrose 1 M is a potent releaser of appetitive responses overcoming even malaise-like situations (Fig. 6). It can, therefore, overcome the physiological distress of aversive substances, probably through the additional supply of energetic resources. On the other hand, in the case of quinine, it has been shown that a mixture of quinine and sucrose solution suppresses the spiking activity of sucrose- receptor neurons [6]. How this suppression relates to the increase in individual survival upon mixture ingestion remains unclear. An interaction in terms of reduced absorption of the toxic compound could occur when mixed with sucrose, thereby reducing the speed of uptake of the toxic compound in the gut. In addition, the ingestion of sucrose may trigger the production of other digestive enzymes that could have an indirect detoxifying role.

### A different scenario: the effect of aversive compounds on freely-moving honeybees

The conclusion that aversive compounds induce a post-ingestional malaise-like state rather than generating gustatory aversion applies to the experimental conditions used in our work, i.e. to the fact that bees were immobilized in individual harnesses and did not have, therefore, the possibility to actively avoid aversive stimulations. This issue is particularly critical, and one has to be cautious before concluding that the effect of aversive compounds found in our experiments is the general mechanism of action of these substances. Indeed, the issue of having an animal constrained or free to move may result in dramatic changes in behavioral performances so that wrong conclusions can be made if one considers just one of these aspects. For instance, comparing color discrimination in harnessed bees and freely-flying bees yields surprisingly different performances [60], [61]. While freely-flying bees are capable of extremely fine discriminations in certain regions of their color spectrum, particularly at the intersection of photoreceptor sensitivity curves [60] where wavelength differences down to 4.5 nm can be discriminated, harnessed bees in the laboratory show extremely poor discrimination power for different wavelengths that they can definitely discriminate when they freely fly and choose among color targets [61]. In the laboratory, bees can be trained to associate a color with sucrose reward so that they extend their proboscis to the learned color after successful training [61], [62]. In this protocol, bees have difficulties in learning this association, and show very poor color discrimination abilities [61]. This difference may be due to motivational factors as cutting the bees' antennae is necessary for the harnessed bees to learn visual cues [62], [63] and it has been recently shown that this procedure substantially decreases the subjective value of sucrose as a reward [64]. The important conclusion that can be derived from these experiments is that concluding that bees have extremely poor color discrimination capabilities based solely on the laboratory experiments with harnessed bees would be a mistake. Similarly, we need to contemplate the possibility that in another experimental scenario, with bees that freely express their choices and avoidance behaviors, the effect of aversive compounds may be different.

Precisely, new experiments on color discrimination by freely-flying bees [30] show that highly concentrated quinine solution (60 mM) exerts strong aversion via gustatory pathway rather than through a malaise-like state. Avargues-Weber et al. [30] studied the capacity of freely-flying bees to discriminate colors that are extremely similar in perceptual terms. One color (the target or CS+) was associated with sucrose 1 M and a different color (the distracter or CS-) was associated either with water for one group of bees (water group), or with concentrated quinine solution for another group of bees (quinine group). The question raised by this work was whether the quinine solution, acting as a negative reinforcer, would improve color discrimination in the quinine group compared to the water group. This was indeed the case as the presence of quinine on the CS- promoted discrimination between CS+ and CS- while the presence of water did not. Moreover, measuring drinking times was possible in these experiments by confronting bees with drops of sucrose solution, water and 60 mM quinine solution. It was shown that when bees have the opportunity to actively express their choice, they explicitly avoided quinine solution. Indeed, average contact time with quinine was extremely reduced (0.7±0.2 seconds) and lower than that measured for water (1.7±0.3 seconds); considering that these times included the extension and retraction of the proboscis, it was concluded that freely-flying bees rejected the quinine solution and that their avoidance was mediated by a gustatory input, i.e. via a distasteful sensory experience, rather than by a post-ingestional malaise.

These results indicate, therefore, that the two experimental scenarios, freely-flying bees and harnessed bees in the laboratory, determine different gustatory performances in honeybees. It would be, nevertheless, critical to confirm this difference in one and the same experiment in order to exclude confounding variables derived from the fact that different results were obtained in different experiments, locations, seasons, etc, and by different experimenters. Such an experiment should test whether the possibility to freely move significantly affects taste preference and taste behavior of honeybees. If this were the case, the fundamental goal to reach would be to determine the kind of physiological switch changing acceptance or rejection thresholds for aversive substances once bees are immobilized.

### Gustatory responses of honeybees in an ecological context

In natural conditions, intoxications leading to malaise should be, in principle, rare. Even if bees consume nectars and/or pollens with high contents of deterrent secondary compounds (e.g., alkaloids, glycosides, phenolic substances), one has to keep in mind that combining deterrent substances such as quinine or LiCl with sucrose solution suppressed mortality in our experiments (see Fig. 2). Thus, although most secondary compounds studied so far actually deter honeybees within a range of high concentrations [65], having them in nectar may reduce considerably their harmful effects. Moreover, natural concentrations of secondary compounds in nectar and pollen are usually much lower, thus decreasing even more their potential impact. For instance, naturally occurring concentrations of amygdalin are between 4 and 10 ppm [66] which correspond to 8.75×10^−6^ M and 2.19×10^−5^ M, respectively. Honeybees seem to cope efficiently with this natural range of concentrations. Whereas high concentrations of phenolic substances deter them [67], low concentrations are attractive to them [24]. Likewise, bees prefer low concentrations of amygdalin during early summer but not later [66]. Some alkaloid-containing nectars attract bees in the field even when alternative nectar sources are available [68]. For instance, honeybees prefer solutions with low concentrations of nicotine and caffeine over a control (20% sucrose) solution [26]. A similar but non-significant pattern was detected also for all concentrations of amygdalin [26]. It seems, therefore, that consistently with our results, nectars containing substances that are considered deterrent due to their unpalatable taste are in fact consumed by honeybees. The fact that mixtures of aversive compounds and sucrose solution do not seem to induce a malaise-like state may indicate that in a natural context such a post-ingestional malaise would rarely occur.

### Perspectives

Our results show that in harnessed bees the ingestion of toxic, aversive substances leads to a post-ingestion malaise-like state that leads the animal to reduce their choice of an odor that was previously learned as appetitive. Despite the fact that such a situation would not necessarily occur in an ecological context, our work has the merit of potentially establishing a new protocol for aversive learning in bees, which could be further developed. Although our experimental design did not exactly follow that used in conditioned taste aversion experiments, it is possible to conceive modifications of our procedure to set equivalent conditioned taste aversion experiments in honeybees. The advantage of such an endeavor would be to dispose of an additional protocol for the study of aversive learning in honeybees. So far, the study of learning and memory in honeybees has focused on a single hedonic modality, the appetitive one, in which bees are rewarded with sucrose solution in different experimental frameworks [36]. Only recently, the integrative study of aversive learning in honeybees, combining behavioral and neural analyses, started to be possible thanks to the establishment of a new conditioning protocol, the olfactory conditioning of the sting extension reflex [69]–[71]. This protocol uses an electric shock as aversive reinforcement (US) which is paired with a neutral odorant (CS). After successful conditioning bees extend the sting to the aversive CS which predicts shock delivery. Combining this protocol with neuropharmacological blocking of aminergic neurotransmitters allowed determining that in honeybees, like in Drosophila [72] and crickets [73], [74], the aversive US is mediated by dopaminergic signaling [69]. We predict that aversive taste experiences, as demonstrated in the experiments with freely-flying bees [30] (see above) are mediated by dopaminergic signaling. Indeed, in crickets, the aversive gustatory stimulation of saline solution [73], [74] is mediated by dopaminergic signaling.

Whether the malaise-like state evinced in our experiments also activates the dopaminergic system to signal a displeasing situation is doubtful. A candidate biogenic amine for this kind signaling could be serotonine (5-hydroxytryptamine, 5-HT), which has been shown to regulate feeding and feeding related processes such as hunger, gut motility and dieresis in numerous insect species (crickets *Teleogryllus commodus*: [75]; migratory locusts *Locusta migratoria* : [76]; fall armyworms *Spodoptera frugiperda*: [77]; cabbage worms *Pieris rapae*: [78]; stick insects *Carausius morosus*: [79], among others). In this way, while in freely-flying bees, the aversive gustatory signaling would be mediated by the dopaminergic system, in harnessed bees, the activation of the serotoninergic system could underlie malaise-like states. If these hypotheses proved to be true, and whatever the signaling strategy used to represent malaise as an aversive US would be, a fundamental goal would be to determine whether there is indeed a perceptual and behavioral switch from the harnessed to the freely-flying bee condition, and the neural changes underlying such a switch.
